# Anticancer Effects and Molecular Mechanisms of Apigenin in Cervical Cancer Cells

**DOI:** 10.3390/cancers14071824

**Published:** 2022-04-04

**Authors:** Ya-Hui Chen, Jyun-Xue Wu, Shun-Fa Yang, Chueh-Ko Yang, Tze-Ho Chen, Yi-Hsuan Hsiao

**Affiliations:** 1 Women’s Health Research Laboratory, Changhua Christian Hospital, Changhua 50006, Taiwan; 106317@cch.org.tw (Y.-H.C.); 183726@cch.org.tw (J.-X.W.); 64420@cch.org.tw (C.-K.Y.); 2 Institute of Medicine, Chung Shan Medical University, Taichung 40201, Taiwan; ysf@csmu.edu.tw; 3 Department of Medical Research, Chung Shan Medical University Hospital, Taichung 40201, Taiwan; 4 Department of Obstetrics and Gynecology, Changhua Christian Hospital, Changhua 50006, Taiwan; 46305@cch.org.tw; 5 School of Medicine, Chung Shan Medical University, Taichung 40201, Taiwan; 6 College of Medicine, Kaohsiung Medical University, Kaohsiung 807378, Taiwan; 7 Department of Post-Baccalaureate Medicine, College of Medicine, National Chung Hsing University, Taichung 40227, Taiwan

**Keywords:** cervical cancer, apigenin, apoptosis, integrin β1-FAK, PI3K/AKT, EMT

## Abstract

**Simple Summary:**

The present study investigated the anticancer effects and molecular mechanisms of apigenin in cervical cancer in vitro and in vivo. HeLa and C33A cells were treated with apigenin; the apigenin inhibited cell viability, induced cell cycle arrest, and inhibited migration and epithelial-to-mesenchymal transition (EMT) of cervical cancer cells. In a cervical tumor xenograft mouse model, apigenin suppressed the growth of C33A xenograft tumors. The apigenin down-regulated FAK signaling (FAK, paxillin, and integrin β1) and PI3K/AKT signaling (PI3K, AKT, and mTOR), which inactivated or activated various signaling targets, such as Bcl-2, Bax, p21^cip1^, CDK1, CDC25c, cyclin B1, fibronectin, N-cadherin, vimentin, laminin and E-cadherin, leading to mitochondrial-mediated apoptosis, G2/M-phase arrest, and reduction in cancer cell migration, thereby producing anticancer effects in cervical cancer. Thus, apigenin may have potential as a chemotherapeutic agent for cervical cancer treatment.

**Abstract:**

Cervical cancer is the fourth most frequent malignancy in women. Apigenin is a natural plant-derived flavonoid present in common fruit, vegetables, and herbs, and has been found to possess antioxidant and anti-inflammatory properties as a health-promoting agent. It also exhibits important anticancer effects in various cancers, but its effects are not widely accepted by clinical practitioners. The present study investigated the anticancer effects and molecular mechanisms of apigenin in cervical cancer in vitro and in vivo. HeLa and C33A cells were treated with different concentrations of apigenin. The effects of apigenin on cell viability, cell cycle distribution, migration potential, phosphorylation of PI3K/AKT, the integrin β1-FAK signaling pathway, and epithelial-to-mesenchymal transition (EMT)-related protein levels were investigated. Mechanisms identified from the in vitro study were further validated in a cervical tumor xenograft mouse model. Apigenin effectively inhibited the growth of cervical cancer cells and cervical tumors in xenograft mice. Furthermore, the apigenin down-regulated FAK signaling (FAK, paxillin, and integrin β1) and PI3K/AKT signaling (PI3K, AKT, and mTOR), inactivated or activated various signaling targets, such as Bcl-2, Bax, p21^cip1^, CDK1, CDC25c, cyclin B1, fibronectin, N-cadherin, vimentin, laminin, and E-cadherin, promoted mitochondrial-mediated apoptosis, induced G2/M-phase cell cycle arrest, and reduced EMT to inhibit HeLa and C33A cancer cell migration, producing anticancer effects in cervical cancer. Thus, apigenin may act as a chemotherapeutic agent for cervical cancer treatment.

## 1. Introduction

Cervical cancer is the fourth most frequent malignancy in women [1]. Human papilloma virus (HPV) infection is the main cause of development of cervical cancer [2]. At present, cervical cancer treatment options include surgical resection, radical therapy, chemotherapy or combination therapy, and immunotherapy. However, the overall 5-year survival rate is only 68% for advanced cervical cancer patients, demonstrating that the treatment remains unsatisfactory [3]. Chemotherapy has been considered a standard treatment for advanced or recurrent cervical cancer patients, but the first-line therapeutic drug cisplatin appears to generate chemoresistance, reducing the therapeutic efficacy [4]. Therefore, elucidating the mechanisms contributing to the malignant progression of cervical cancer and developing novel therapy agents are very important.

Apigenin is a natural plant-derived flavonoid (4′,5,7-trihydroxyflavone), which belongs to flavone group of glycosides. It is present in common fruit (oranges and grapefruit), vegetables (onions and parsley) and herbs (chamomile and basil) [5]. Apigenin has been reported to act as a free-radical scavenger with antioxidant, anti-inflammatory, anti-mutagenic, anti-hyperglycemic, and antiviral effects [6,7,8,9]. In addition, apigenin has been shown to increase the activities of intracellular glutathione reductase (GSH) and superoxide dismutase (SOD), enhancing the endogenous defense against oxidative stress [10]. Several studies have demonstrated that apigenin acts as an anticancer agent in various human cancers, both in vivo and in vitro, such as breast, prostate, ovarian, lung, liver, pancreatic, and colon cancers [11,12,13,14,15,16,17], in addition to cervical cancer [18,19]. Moreover, apigenin has been demonstrated to mediate anticancer effects via molecular mechanisms potentially involving caspase-3, -8, Bax, and TNF-α activation; Bcl-2, MMP-2, -9, Snail, and Slug inactivation; decreased NF-κB, PI3K, AKT, phospho-AKT, p38, MAPK, ERK, and JNK expressions; and activated proteasomal Her2/neu protein degradation [5,20,21].

Strouch et al. [22] and Hu et al. [23] revealed that when apigenin is combined with gemicitabine or 5-FU, it can effectively inhibit cancer cell proliferation and tumor progression to a greater degree than either agent alone in pancreatic cancer and hepatocellular carcinoma, respectively. Kim et al. [24] also indicated that apigenin combined with the targeted therapy, PLX4032 (BRAFV600E inhibitor), synergistically inhibits thyroid carcinoma cell viability; the protein levels of cleaved PARP1 and cleaved caspase-3 were elevated, and phospho-ERK and phospho-AKT were reduced as compared with therapy with either agent alone. Furthermore, apigenin has been investigated in several clinical trials involving Alzheimer’s disease [25], insomnia [26], anxiety disorder [27], knee osteoarthritis [28], and depression [29], and the results indicated that apigenin could improve brain cognitive performance, provide modest improvement in daytime functioning, reduce demand for analgesics, reduce anxiety disorder symptoms, and lower the score on the Hamilton depression rating scale. Although apigenin is known as a health-promoting and anticancer agent, its use in chemotherapy in various cancers is not widely accepted by clinical practitioners, and thus the beneficial anticancer effects of apigenin need identifying, with more precise mechanisms ascertained via in vitro and in vivo studies.

The evidence has indicated that the epithelial-to-mesenchymal transition (EMT) is a major process associated with cancer cells, rendering migration and invasion easier, reducing epithelial cell intercellular adhesion, and increasing cell motility, resulting in colonization and metastases formation by cancer cells [30,31,32]. Thiery et al. [33] revealed that EMT can contribute to cancer stem cell generation of immune suppression, increased resistance to apoptosis and senescence, and development of therapy resistance in cancer cells, such as tamoxifen-resistant breast MCF-7 cancer cells [34] and gemcitabine-resistant pancreatic tumor cells [35]. Previous study has also demonstrated that EMT is implicated in poor cervical cancer prognoses through inactivation of E-cadherin and activation of vimentin [36]. Thus, in the present study, we investigated the effects of apigenin against cervical cancer and studied the underlying mechanisms, using both cervical cancer cells (HeLa, C33A) and a xenograft mouse model.

## 2. Materials and Methods

### 2.1. Cell Culture

Human cervical cancer (HeLa and C33A) cell lines were purchased from the Bioresource Collection and Research Centre (BCRC, Hsin-Chu, Taiwan; derived from ATCC CRM-CCL-2 and ATCC CRM-HTB-31) and cultured in Minimal Essential Medium Alpha ((#12571-063, MEM α, Thermo Fisher Scientific Inc., Waltham, MA, USA), supplemented with 10% fetal bovine serum (TMS-013-BKR, Sigma-Aldrich, St. Louis, MO, USA) and penicillin (10 IU/mL)/streptomycin (10 mg/mL) (#15140-122, Thermo Fisher Scientific Inc.) at 37 °C in a humidified incubator with 5% CO_2_.

### 2.2. Assay of Cell Viability

Cells were seeded into 96-well plate at a density of 1 × 10^4^ cells per 100 μL culture medium for 24 h. After cell attachment, culture medium with apigenin at varying concentrations (0, 1, 10, 25, 50, 100 μmol/L) in dimethyl sulphoxide (D26650, DMSO, Sigma-Aldrich) was used to treat the cells for 24 h. Apigenin (≥99.22% purity by LCMS, Figure S1) was procured from MedChem Express (#HY-N1201, Monmouth, NJ, USA). At the end of incubation, the medium was discarded, and the cells were washed with Dulbecco’s phosphate-buffered saline (#14190-144, DPBS, pH 7.4, Thermo Fisher Scientific Inc.) twice. To each well, 90 μL fresh culture medium and 10 μL presto/blue (A13262, Thermo Fisher Scientific Inc.) were added. After 4 h of incubation at 37 °C, the absorbance of the samples in the plates was measured at 570 nm with a reference wavelength set at 600 nm for PrestoBlue, using a microplate reader (Thermo Fisher Scientific, MA, USA). Cells treated with the vehicle control (0.1% DMSO in the culture medium) were regarded as 100% viable, and the viability of the apigenin-treated cells was determined.

### 2.3. Assay of Cell Cycle Progression

Cells were treated with 50 μM apigenin or vehicle control for 24 h, then harvested with trypsin, washed twice with DPBS, and fixed in ice-cold 70% ethanol overnight at 4 °C. The cells were then washed with ice-cold DPBS twice and incubated with 25 μL propidium iodide (20 μg/mL, #1056-1, BioVision, Inc., Waltham, MA, USA) and 10 μL DNase-free RNase A (10 mg/mL, RA02, GeneMark, Taipei, Taiwan) for 30 min at 37 °C in the dark. Lastly, the stained cells were analyzed using FC500 flow cytometry (Beckman Coulter, Brea, CA, USA). The percentages of cells in different cell cycle stages (Sub G1, G0/G1, S, and G2/M phases) were calculated using CXP software (ver. 2.3, Beckman Coulter, Brea, CA, USA).

### 2.4. Wound-Healing Migration Assay

HeLa and C33A cancer cells were seeded onto 6-well plates and grown to 70–80% confluence. Straight wounds were made by using a 200 μL sterile tip to create a scratch in the center of the monolayer cells. After washing with medium to remove non-adherent cells, the wounded monolayers were treated with or without 50 μM apigenin, and images of the wound gaps were obtained under an Olympus BX61 microscope (Tokyo, Japan) at 0, 24, and 48 h. The wound areas were quantitatively evaluated using ImageJ software (http://rsb.info.nih.gov/ij/, accessed on 1 December 2020, NIH, Bethesda, MD, USA). To reduce variability in the results, multiple views of each well were documented, and each group experiment was repeated at least three times.

### 2.5. Western Blot Analysis

The protein concentrations of subcellular extracts were quantitated by BCA assay (#23225, Thermo Fisher Scientific Inc.); 25 μg of protein were loaded and separated by 8–12% (*w*/*v*) sodium dodecyl sulfate-polyacrylamide gel electrophoresis, then transferred onto a 0.2-μm polyvinylidene difluoride (PVDF, EA162-0177, Bio-Rad, Irvine, CA, USA) membrane. The membranes were then blocked with 0.5% bovine serum albumin (BSA, A3294, Sigma-Aldrich) in PBS/0.5% Tween 20 (#9005-64-5, Sigma-Aldrich) for 1 h, followed by incubation with primary antibodies (1/800-1/1000; diluted in blocking buffer) overnight at 4 °C: CDK1 (E-AB-64159), CDC25c (E-AB-63512), cyclin B1 (E-AB-70114), p21^cip1^ (E-AB-65412), Bcl-2 (E-AB-15522), and Bax (E-AB-30629e) purchased from Elabscience Technology (Houston, TX, USA); phospho-AKT (#4060S), AKT (#9272S), phospho-mTOR (#2971S), and mTOR (#2972S) (Cell Signaling Technology; Danvers, MA, USA); phospho-FAK (GTX129840), FAK (GTX100764), paxillin (GTX129840), and integrin β1 (GTX128839) (GeneTexT Biotechnology; Hsinchu city, Taiwan); PI3K(p85) (ARG55392), fibronectin (ARG66162), N-cadherin (ARG22587), vimentin (ARG66199), laminin (ARG59198), and E-cadherin (ARG66195) (Arigo Biolaboratories Biotechnology; Hsinchu city, Taiwan); phospho-PI3K(p85) (AB182651; Abcam Technology; Cambridge, UK) and GAPDH (MA5-15738; Thermo Fisher Scientific Inc.). After washing, the membranes were incubated with HRP-conjugated secondary antibodies (1/10,000 diluted in blocking buffer; Mouse#115-035-003; Rabbit#111-035-003; Jackson ImmunoResearch, Laboratories, Inc. West Grove, PA, USA) for 1 h at room temperature. Signals were detected by an enhanced chemiluminescence reagent (K-12045-D50, ECL, Advansta Inc., San Jose, CA, USA) and visualized using the Fusion FX7 image system (Vilber Lourmat, Marne-la-Vallée Cedex, France). Bands were quantified using ImageJ^TM^ software (NIH) and normalized to GAPDH.

### 2.6. Human Cervical Tumor Xenograft Mouse Model

Twelve female BALB/c mice, aged 7 weeks, were purchased from the National Laboratory Animal Center (Taipei, Taiwan) and randomly assigned into two groups (*n* = 6 in each group). All animals were bred in a specific pathogen-free conditional house and 12:12 h light/dark cycle at 22 °C. The animal experiment protocols were approved by the Institutional Animal Care and Use Committee (IACUC) of the Changhua Christian Hospital, Taiwan (approval no: CCH-AE-108-013). Human cervical cancer C33A cells (1 × 10^7^) and Matrigel reagent (#354248, Corning Inc., Tewksbury, MA, USA) were mixed (cells: Matrigel = 2:1) and injected subcutaneously into the right flank of each mouse. Once the tumor volume reached ~200 mm^3^ (Day 9), the mice were treated with apigenin [50 mg/kg, dissolved in 10% DMSO, 40% Cremophor/ethanol (3:1; #C5135, Sigma-Aldrich), and 50% PBS] or vehicle [10% DMSO, 40% Cremophor/ethanol (3:1) and 50% PBS] by intraperitoneal injection every day for 16 days (Day 25). Tumor size (measured using an electronic caliper) and mouse body weight were recorded every two days, and tumor volumes were calculated using the following standard formula: length × width^2^/2. At the end of the experiment, the tumors were collected and extracted for tissue analysis. The cancer cell implantation was conducted using 2–3% isoflurane (Panion & BF Biotech Inc., Taipei, Taiwan) inhalation, and the mice sacrifice used a CO_2_ chamber.

### 2.7. Histology and Immunohistochemical Analysis

C33A xenograft fresh tumor tissue was fixed with 10% neutral buffered formalin (#3800600, Leica Biosystems Richmond, Inc., Richmond, IL, USA). embedded in paraffin, and then cut into 5-μm sections. Briefly, all samples were examined histologically after hematoxylin and eosin staining (#3801698, Leica Biosystems Richmond, Inc.). The paraffin-embedded tissues were deparaffinized, rehydrated, and washed in PBS. To block non-specific binding, sections were incubated with 3% BSA (A3294, Sigma-Aldrich, in PBS) for 1 h. Sections were further incubated with primary antibodies against Ki67 (#12202; 1/400, Cell Signaling), Bcl-2 (#15071; 1:400, Cell Signaling), cyclin B1 (E-AB-70114; 1:300, Elabscience), phospho-FAK (GTX129840; 1/200, GeneTex), paxillin (GTX129840; 1/100, GeneTex), integrin β1 (GTX128839; 1/100, GeneTex), fibronectin (ARG66162; 1/200, Arigo Biolaboratories), N-cadherin (ARG22587; 1/100, Arigo Biolaboratories), vimentin (ARG66199; 1/500, Arigo Biolaboratories), laminin (ARG59198; 1/500, Arigo Biolaboratories), and E-cadherin (ARG66195; 1/50, Arigo Biolaboratories) in PBS overnight at 4 °C. Sections on slides were washed with PBS and incubated with OneStep Polymer HRP-conjugated anti-mouse/rat/rabbit IgG secondary antibody (GTX83398; GeneTex Biotechnology; Hsinchu city, Taiwan) for 30 min at room temperature. The peroxidase activity was visualized with a 3,3′-diaminobenzidine chromogent reagent (DAB; GTX30939; GeneTex) counterstained with hematoxylin. Images were obtained using an Olympus BX61 microscope (Olympus Corporation, Shinjuku, Tokyo, Japan), and the results were determined by counting the numbers of positive cells in four fields of specimens from each group by Image-Pro Plus 4.5 software (Media Cybermetics, Silver Spring, MD, USA).

### 2.8. Statistical Analysis

Statistical analysis was performed using a Student’s *t* test (for two-group comparison, Microsoft Excel 2016, Microsoft, Washington, DC, USA). Data are reported as means ± standard deviation (SD) and all data represent the results of at least three independent experiments. *p* < 0.05 was considered significant.

## 3. Results

### 3.1. Apigenin Inhibits Human Cervical Cancer Cell Viability and Induces Cell Cycle Arrest

**Figure 1:**
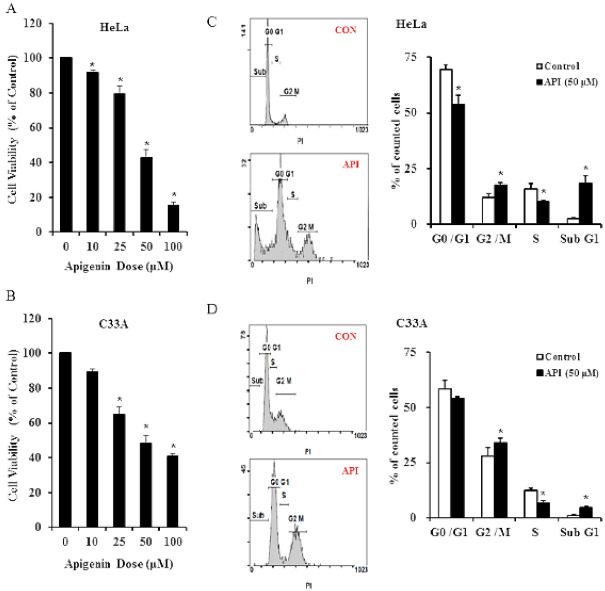
Apigenin inhibits human cervical cancer cell viability and induces cell cycle arrest. HeLa (**A**) and C33A (**B**) cells were treated with increasing doses of apigenin for 24 h. Cell viability was determined using PrestoBlue™ cell viability reagent. HeLa (**C**) and C33A (**D**) cells were treated with or without 50 μM of apigenin for 24 h, and an estimation of the cell cycle phase distribution (G0/G1, S, and G2/M) was determined by PI staining via flow cytometry, followed by quantification. Data are presented as the mean ± SD of at least three independent experiments. * *p* < 0.05 indicates a significant difference as compared with the corresponding control. CON, 0.1% DMSO; API (50 μM), 50 μM apigenin.

We assessed the effect of apigenin on the viability of human cervical cancer cells. A PrestoBlue assay was performed on HeLa and C33A cells, and cells were exposed to varying concentrations of apigenin (0–100 μM). We observed that apigenin had a cytotoxic effect on cells and inhibited cell growth (reducing cell viability) in a dose-dependent manner. After 24 h of treatment, 50 µM apigenin inhibited HeLa and C33A cell growth by 52.5–61.6% and 46.1–58.6%, respectively. Thus, we chose to employ this dose in other experiments. A higher dose of apigenin (100 μM) resulted in a lower cell viability for HeLa cells, but the viability of C33A cells did not change significantly. These data demonstrated clearly that apigenin exerts inhibitive effects on cervical cancer cell growth (Figure 1A,B, *p* < 0.05). To understand whether apigenin affects cell cycle progression of cervical cancer cells, the distribution of cells in the different cell cycle phases was evaluated by flow cytometry. For HeLa cells, apigenin demonstrated significant inhibitive effects on cell cycle progression arresting at the G0/G1 and S phases (G0/G1: 69.64% → 53.86%; S: 15.90% → 10.39%), and the numbers of cells were significantly increased in the sub G1 and G2/M phases (G2/M: 11.95% → 17.44%; sub G1: 2.5% → 18.31%) as compared with the vehicle control group (Figure 1C, *p* < 0.05). Regarding C33A cells, apigenin also significantly reduced the proportion of cells in the S phase and increased the ratio of cells in the sub G1 and G2/M phases (Figure 1D), but the change was less obvious than for HeLa cells. These results demonstrated that apigenin-induced cervical cancer cell death is mediated by cell cycle arrest.

### 3.2. Apigenin Causes G2/M Phase Arrest by Modulating Cyclin B1/CDK1 and p21^cip1^ as Well as Activating Mitochondrial-Mediated Apoptosis

**Figure 2:**
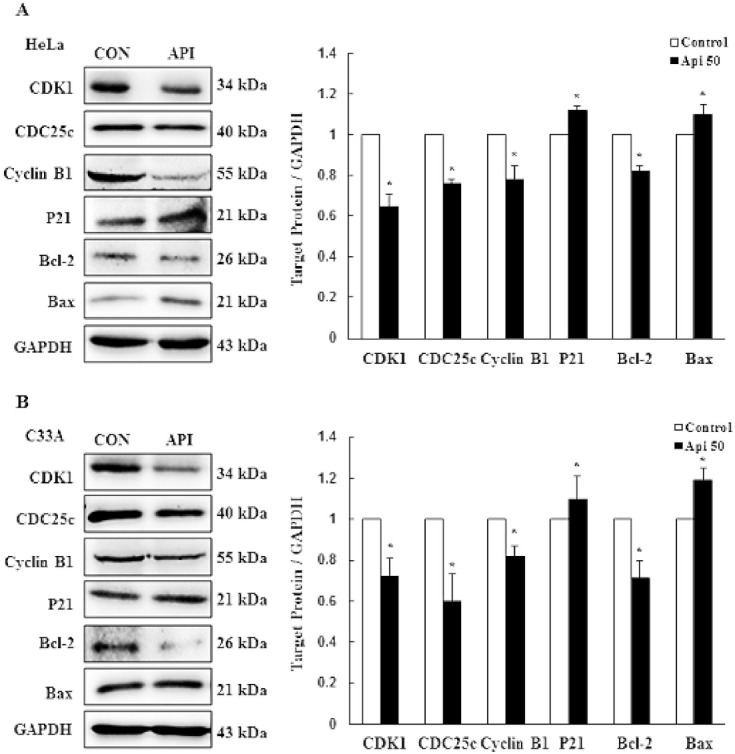
Proposed mechanism and signaling pathways of the apoptosis and cell cycle arrest induced by apigenin in cervical cancer cells. Cell cycle G2/M phase-related proteins CDK1, CDC25C, cyclin B1, and p21, and apoptosis-related proteins, Bcl-2 and Bax, were detected in HeLa (**A**) and C33A (**B**) cells with or without 50 μM apigenin treatment for 24 h via Western blotting and quantified. Data are presented as the mean ± SD of at least three independent experiments. * *p* < 0.05 indicates a significant difference as compared with the corresponding control. CON, 0.1% DMSO; Api 50, 50 μM apigenin.

To further clarify the underlying mechanism responsible for how apigenin affects cell growth in cervical cancer cells, the expression levels of related proteins were examined by Western blotting after treatment with 50 μM apigenin for 24 h. With regards to cell cycle-regulating proteins, including CDK1, CDC25c, cyclin B1 and p21^cip1^, not only HeLa, but also C33A cells were regulated by apigenin. As expected, apigenin significantly inhibited the protein levels of CDK1, CDC25c, and cyclin B1 in HeLa and C33A cells. Relatively, the cell cycle inhibitory protein p21^cip1^ was significantly up-regulated in apigenin-treated HeLa and C33A cells. To further demonstrate that the anti-proliferative effect of apigenin is also due to the initiation of apoptosis, we examined apoptosis-related proteins Bcl-2 and Bax by Western blotting, which demonstrated that apigenin significantly reduced the amount of Bcl-2 and significantly elevated Bax in both HeLa and C33A cells (Figure 2 and Figure S2, *p* < 0.05). These results demonstrated that apigenin caused G2/M phase arrest and apoptosis of HeLa and C33A cells through cyclin B1/CDK1 and p21^cip1^, and activated the mitochondrial-mediated pathway.

### 3.3. Apigenin Induces Cytotoxicity and Apoptosis via the PI3K/AKT/mTOR Pathway

**Figure 3:**
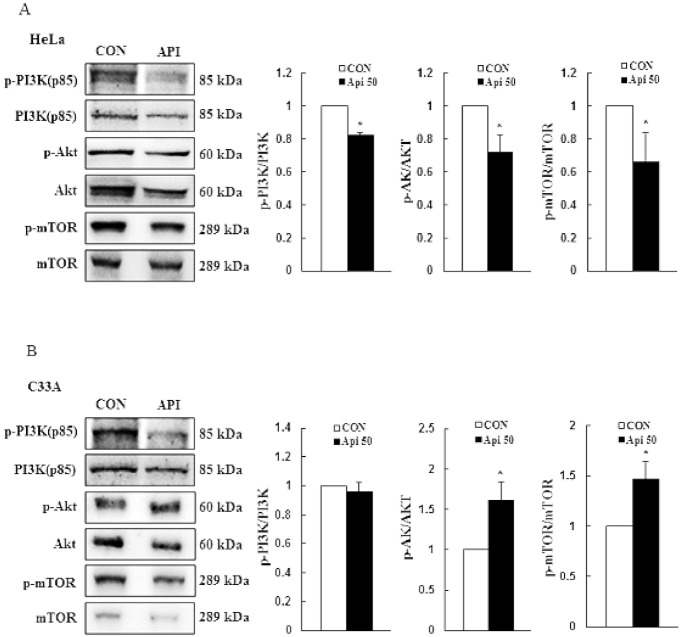
Effects of apigenin on the PI3K/AKT/mTOR signaling pathway in cervical cancer cells. Proteins p-PI3K (p85), PI3K (p85), p-AKT, AKT, p-mTOR, and mTOR were detected in HeLa (**A**) and C33A (**B**) cells with or without 50 μM apigenin treatment for 24 h via Western blotting and quantified. Data are presented as the mean ± SD of at least three independent experiments. * *p* < 0.05 indicates a significant difference as compared with the corresponding control. CON, 0.1% DMSO; Api 50, 50 μM apigenin.

In order to understand the molecular mechanism of apigenin-induced cytotoxicity and apoptosis in HeLa and C33A cells, we examined phosphorylation of the PI3K/AKT/mTOR pathway by Western blotting. As compared with the control group, apigenin significantly inhibited the phosphorylation levels of PI3K (−0.2 fold), AKT (−0.3 fold), and mTOR (−0.3 fold) in HeLa cells (Figure 3A, *p* < 0.05), whereas apigenin elevated the p-AKT (+0.6 fold) and p-mTOR (+0.5 fold) expression levels in C33A cells but PI3K did not to significantly change (Figure 3B and Figure S3). Thus, these results demonstrated that the PI3K/AKT/mTOR pathway might be involved in apigenin-induced cytotoxicity and apoptosis in human cervical cancer.

### 3.4. Apigenin Inhibits Cancer Cell Migration and Epithelial-to-Mesenchymal Transition (EMT) of Human Cervical Cancer

**Figure 4:**
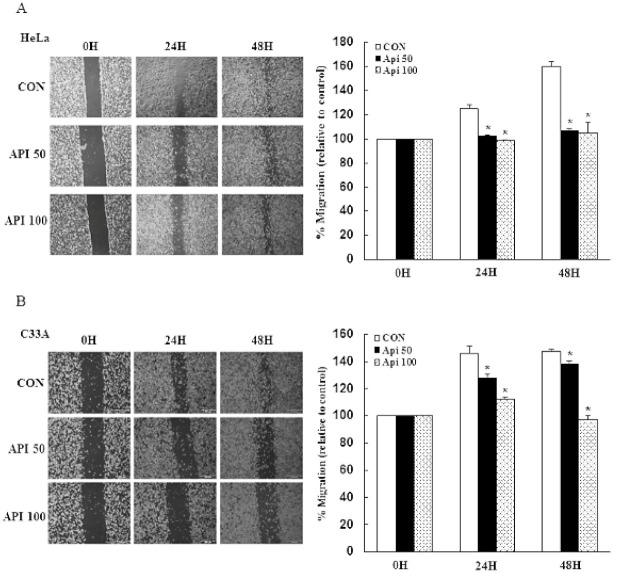

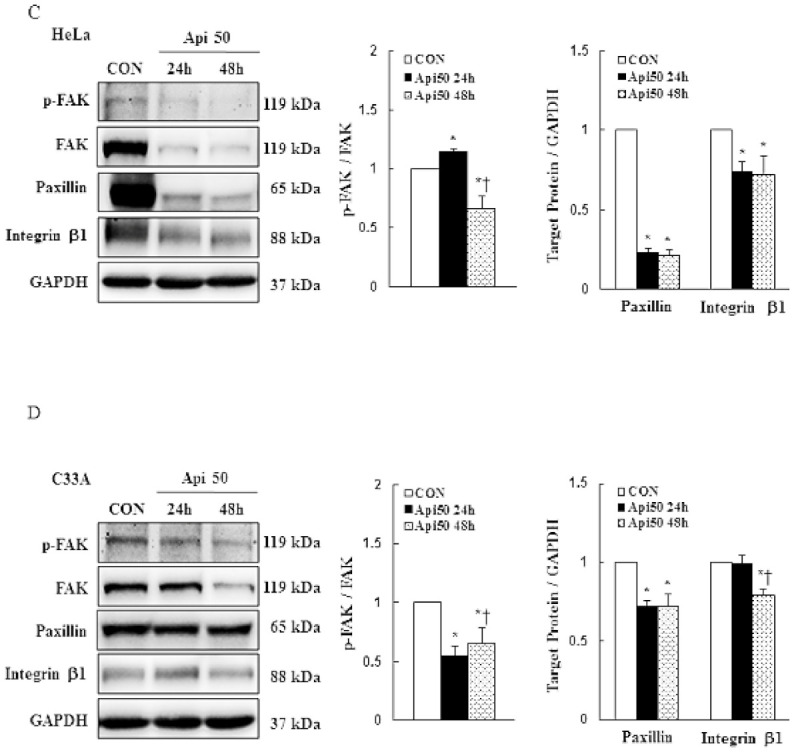
Apigenin inhibits cancer cell migration and inactivates the integrin β1-FAK signaling pathway. Wound-healing assays were performed with or without 50–100 μM apigenin in HeLa (**A**) and C33A (**B**) cells for 0, 24, and 48 h. Left: representative images of scratches and recovery of wounded areas on cell monolayers at 0, 24, and 48 h after wounding. Right: semi-quantitative analysis of relative cell migration was performed according to the cells moving towards the scratched area at a certain time. Cell migration-related proteins p-FAK, paxillin, and integrin β1 were detected in HeLa (**C**) and C33A (**D**) cells with or without 50 μM apigenin treatment for 24 and 48 h via Western blotting and quantified. Data are presented as the mean ± SD of at least three independent experiments. * and † *p* < 0.05 indicate significant differences as compared with the corresponding control or Api 50-treated groups. CON, 0.1% DMSO; Api 50, 50 μM apigenin; Api 100, 100 μM apigenin; Api50 24 h, 50 μM apigenin at 24 h; Api50 48 h, 50 μM apigenin at 48 h.

**Figure 5:**
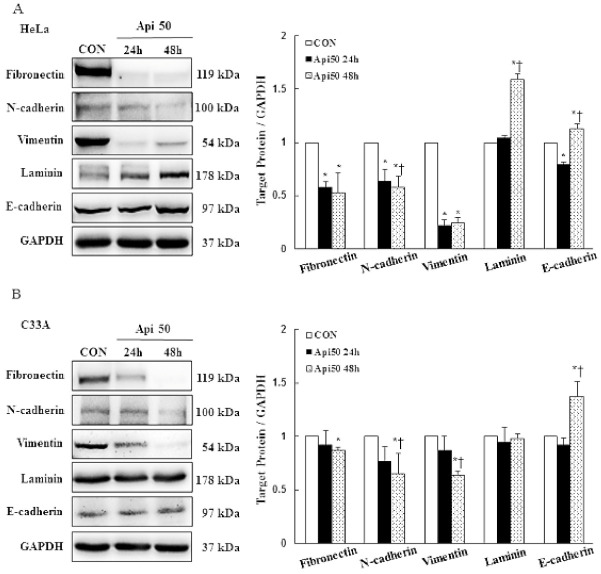
Apigenin disrupts cancer cell metastasis and inhibits epithelial-to-mesenchymal transition. Proteins fibronectin, N-cadherin, vimentin, laminin, and E-cadherin were detected in HeLa (**A**) and C33A (**B**) cells with or without 50 μM apigenin treatment for 24 and 48 h via Western blotting and quantified. Data are presented as the mean ± SD of at least three independent experiments. * and † *p* < 0.05 indicate significant differences as compared with the corresponding control or Api 50-treated groups. CON, 0.1% DMSO; Api50 24 h, 50 μM apigenin at 24 h; Api50 48 h, 50 μM apigenin at 48 h.

To determine whether apigenin treatment affects cancer cell migration and metastasis, we performed in vitro wound-healing and modulation of EMT-related protein assays in HeLa and C33A cells. Our results demonstrated that cells in the vehicle control group had a higher cell migration ability, as their wound closure speed was faster than that of cells treated with 50–100 μM apigenin. Apigenin effectively inhibited cell migration of HeLa and C33A cells as compared with the control group at 24 and 48 h (Figure 4A,B, *p* < 0.05). Due to early studies revealing that FAK acts as strong contributor to the cancer hallmarks in various human cancers, it was activated by integrins; interaction with paxillin resulted in focal adhesion formation and cytoskeleton remodeling promoted tumor invasion and metastasis [37,38,39]. Thus, the integrin/FAK/paxillin signaling was investigated to determine whether the apigenin-inhibited cancer cell migration inactivated the integrin/FAK/paxillin signaling pathway. In the present study, apigenin significantly decreased the phospho-FAK (−0.3 fold), paxillin (−0.8 fold), and integrin β1 (−0.3 fold) protein expression levels in HeLa cells at 48 h, while for C33A cells, the expressions of phospho-FAK, paxillin, and integrin β1 were reduced (−0.3, −0.3, and −0.2 fold, respectively, Figure 4C,D and Figure S4, *p* < 0.05). The expressions of EMT markers in HeLa and C33A cells were altered by apigenin treatment at 50 μM. Apigenin significantly decreased the expressions of fibronectin, N-cadherin, and vimentin (−0.5, −0.4, and −0.8 fold, respectively), while up-regulating the expressions of laminin and E-cadherin (+0.6 and +0.1 fold, respectively) in HeLa cells at 48 h. Similarly, apigenin significantly inhibited the expressions of fibronectin, N-cadherin, and vimentin (−0.2, −0.4, and −0.4 fold, respectively) and significantly enhanced the E-cadherin expression (+0.4 fold), while no change in the expression of laminin was observed, as compared with the non-treated control cells in C33A cells (Figure 5 and Figure S5, *p* < 0.05). Taken together, these results clearly indicated that apigenin plays an important role in terms of disrupting cell migration and cell metastasis, because the integrin β1-FAK signaling pathway and EMT were decreased in human cervical cancer.

### 3.5. Apigenin Suppresses the Growth of C33A Xenograft Tumors

**Figure 6:**
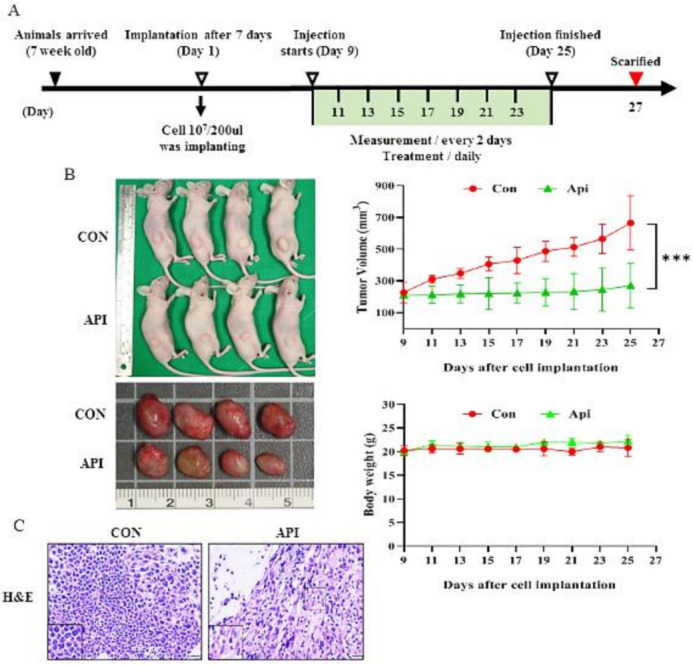

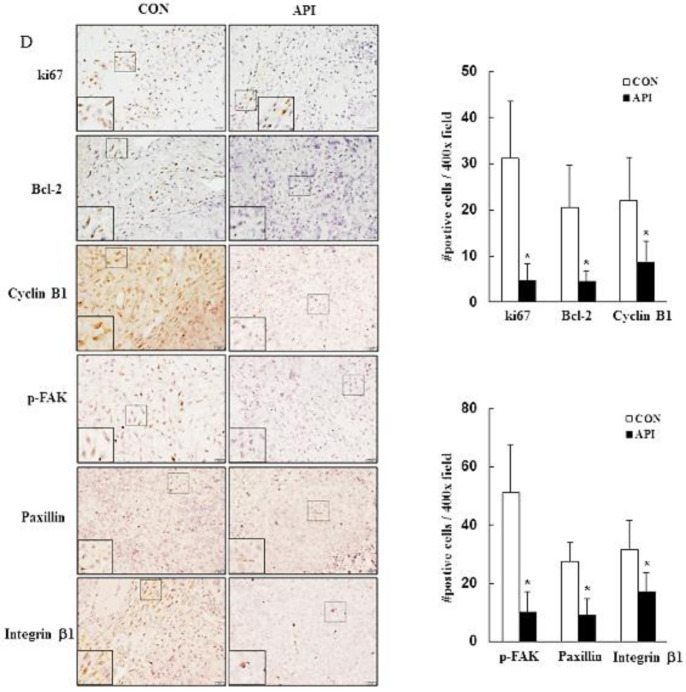

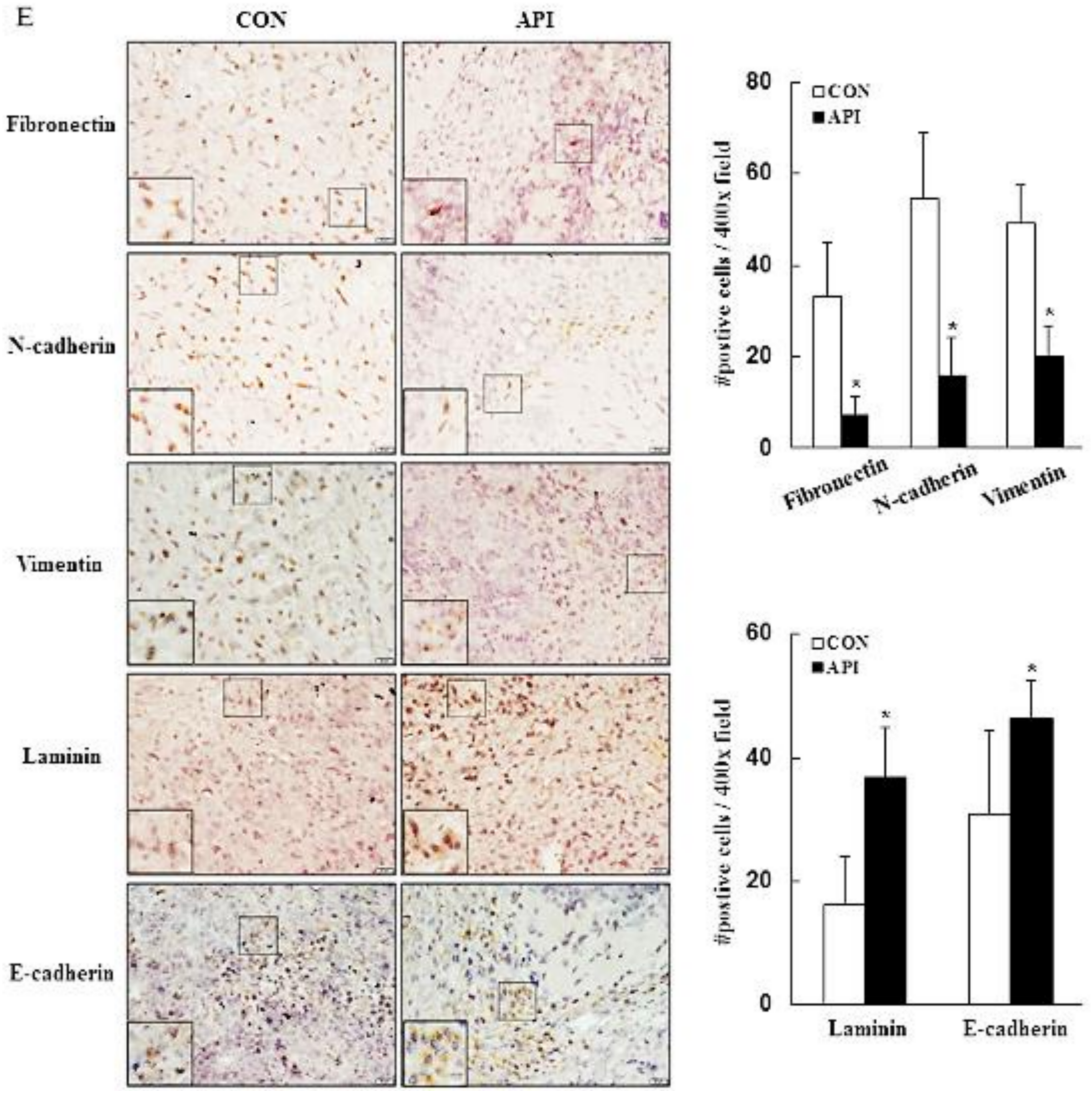
Apigenin suppresses the growth of C33A xenograft tumors in vivo. C33A human cervical cancer cells (1 × 10^7^ cells) were implanted into the right flank of BALB/c nude mice. When the subcutaneous tumor volume reached ~200 mm^3^, mice were treated with the solvent control (10% DMSO) or apigenin (IP, 50 mg/kg/day) for 16 days. (**A**) Schematic representation of the experiment. (**B**) Representative image of a tumor, and average tumor volume and body weight. Tumor tissue samples were analyzed by hematoxylin, eosin staining (**C**), and immunohistochemistry (**D**,**E**) to examine the histopathology and expression levels of ki67, Bcl-2, cyclin B1, phospho-FAK, paxillin, integrin β1, fibronectin, N-cadherin, vimentin, laminin, and E-cadherin (shown as brown staining) (H&E, 400×, bar = 20 μm; IHC, 400×, bar = 20 μm). Values represent the mean ± SD (*n* = 6); * *p* < 0.01, *** *p* < 0.001 indicate significant differences as compared with the corresponding control. CON, control; API, apigenin.

To further confirm the in vitro findings, we investigated the effects of apigenin in a C33A xenograft tumor model using BALB/c nude mice. As per the schematic timeline of this study, as shown in Figure 6A, our data demonstrated that apigenin significantly inhibited the tumor growth of C33A xenografts. After treatment for 16 days, the average tumor volume of the C33A xenograft tumors was 666.0 ± 171.4 and 271.0 ± 138.9 mm^3^ in the control group and apigenin-treated group, respectively. There was no significant difference in body weight between the control and apigenin-treated animals and the final sample size (*n* = 6/group) was a 100% survival rate, suggesting that apigenin did not induce a high host toxicity at a therapeutic dose (Figure 6B, *p* < 0.05). Moreover, the apigenin-treated group demonstrated a significant induced sparse tumor cellularity and apoptosis to tumor tissues as compared with the control (Figure 6C). Furthermore, the immunohistochemistry study demonstrated significantly decreased expressions of ki67 (−5.6 fold), Bcl-2 (−3.6 fold), and cyclin B1 (−1.6 fold) in the apigenin-treated tumors. Consistently, in vitro Western blotting, the analysis demonstrated decreased accumulation of p-FAK (−4.0 fold), paxillin (−2.0 fold), integrin β1 (−0.8 fold), fibronectin (−3.5 fold), N-cadherin (−2.5 fold), and vimentin (−1.5 fold) proteins and a trend of increased laminin (+1.3 fold) and E-cadherin (+0.5 fold) protein expressions in tumors treated with apigenin (Figure 6D,E, *p* < 0.05). These results further indicated that apigenin acted as an anti-proliferative, anti-migratory, and anti-metastatic agent in vivo.

## 4. Discussion

This study revealed the effects of apigenin on cervical cancer cells, including inhibiting cervical cancer cell viability, inducing cell cycle arrest at the G2/M phase by modulating cyclin B1/CDK1 and p21^cip1^, activating mitochondrial-mediated apoptosis, and inhibiting migration and EMT of cervical cancer cells. In a C33A xenograft tumor model, apigenin suppressed the growth of C33A cells.

Our study demonstrated the human cervical cancer cell HeLa, C33A viability, which was consistent with previous studies demonstrating that apigenin caused the cell cycle arrest in the G2/M of head and neck cancer SCC25 [40], colon cancer HCT116 [41], prostate cancer 22Rv1 and PC-3 [42], and breast cancer MDA-MB231 [43], by upregulating the expression of p21^cip1^ and reducing cyclin A/B in MDA-MB231; in addition, apigenin inactivates CDK1 in SCC25 cells, leading to the G0/G1 arrest [44] and further inhibits the apoptosis of SCC25 and MDA-MB231 via the Bcl-2-mediated caspase-dependent cell death pathway.

The PI3K/AKT/mTOR pathway is vital for normal basic cellular function to coordinate cell activities such as proliferation and growth [45,46]. It is one of the most frequently activated signaling pathways, and is aberrantly dysregulated in human cancers; therefore, this pathway is an important pathway for targeted cancer therapy using small molecule inhibitors [45]. In our study, the apigenin induced cytotoxicity and apoptosis via the PI3K/AKT/mTOR pathway. Consistent with the previous reports that demonstrated apigenin’s anti-cancer and chemopreventive effects at cellular and molecular levels, particularly inhibition of the PI3K/AKT/mTOR signaling pathways, the report demonstrated targeting of the PI3K/AKT/mTOR axis by apigenin for cancer prevention [46]. 

Epithelial-mesenchymal transition (EMT) is a cellular program, remolding cell–cell and cell–extracellular matrix interactions. In the process of EMT, epithelial cells detach from each other and the underlying basement membrane [47]. Furthermore, EMT is involved in cancer progression, as well as initiation [48]. Therapeutic control of EMT may contribute to the prevention of cancer metastasis [49]. Several studies have demonstrated that EMT plays a key role in tumor progression in various cancer types, such as pancreatic cancer [50], lung cancer [51], hepatocellular carcinoma [52], and bladder cancer [53].

Focal adhesion kinase (FAK), a tyrosine kinase, can regulate the biological behaviors of tumor cells, such as adhesion, migration, invasion, proliferation, and survival [54], and integrin β1 is important in the development of cervical cancer. The increase in the expression of integrin β1 protein is consistent with the occurrence of lymph node metastasis [55]; the activation of the integrin beta1/FAK signaling pathway is related to cancer metastasis, and the targeting of integrin β1 can attenuate lung cancer metastasis [56]. Apigenin may prevent melanoma metastasis by inhibiting cell migration and diminishing FAK and ERK 1/2 activities. The effects of apigenin on A2058 and A375 melanoma cells have been evaluated [57], consistent with our studies, apigenin effectively inhibited the cell migration of HeLa and C33A cells via inactivation of the FAK signaling (FAK, paxillin, and integrin β1) pathways. In our study, apigenin significantly decreased the expressions of fibronectin, N-cadherin, and vimentin, while increasing the expressions of laminin and E-cadherin in HeLa cells.

Table 1 presents details of previous studies related to the anticancer effect of apigenin on various human cancers, such as leukemia, liver, stomach, brain, cervical, colon, breast, prostate, or oral cancer. These differing molecular mechanisms of the apigenin anticancer effect in various human cancers may be associated with cell line specificity, animal type, and individual apigenin bioavailability (dose- or time-stimuli manner).

## 5. Conclusions

**Figure 7:**
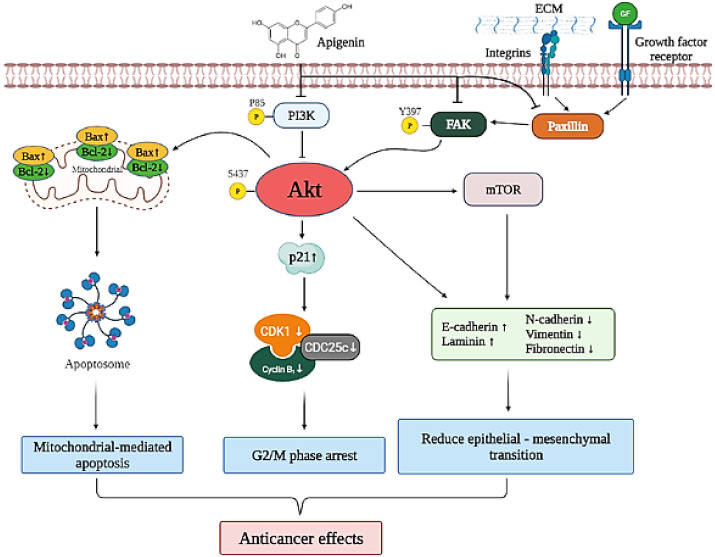
Schematic representation of the anticancer molecular mechanism of apigenin in cervical cancer. Apigenin down-regulated FAK signaling (FAK, paxillin, and integrin β1) and PI3K/AKT signaling (PI3K, AKT, and mTOR), which inactivated or activated various signaling targets, such as Bcl2, Bax, p21^cip1^, CDK1, CDC25c, cyclin B1, fibronectin, N-cadherin, vimentin, laminin, and E-cadherin, leading to mitochondrial-mediated apoptosis, G2/M-phase arrest, and reduced EMT to induce anticancer effects in cervical cancer.

This study demonstrated the multiple anticancer effects of apigenin on cervical cancer cells. The molecular mechanism of apigenin in cervical cancer treatment included down-regulated FAK signaling (FAK, paxillin, and integrin β1) and PI3K/AKT signaling (PI3K, AKT, and mTOR), which inactivated or activated various signaling targets, such as Bcl2, Bax, p21^cip1^, CDK1, CDC25c, cyclin B1, fibronectin, N-cadherin, vimentin, laminin, and E-cadherin, leading to mitochondrial-mediated apoptosis and G2/M-phase arrest, and reduced EMT to result in anticancer effects on cervical cancer (Figure 7). Apigenin may be a potential anticancer treatment modality, and further studies are needed to enable the development of clinical treatment strategies using apigenin against cervical cancer.

## Figures and Tables

**Table 1:** Anticancer effects of apigenin on different human cancers.

Cancer Type	Molecular Mechanism and Activity	Refs.
Leukemia	Apigenin inhibits HL60 cell proliferation via G2/M phase arrest, but TF1 cell was G0/G1 phase arrest	[58]
Liver cancer	Apigenin (5–20 μg/mL) inhibits hepatoma Huh7 cell growth via G2/M phase arrest and apoptosis; Apigenin (50 μg/day) significantly suppressed the growth of Huh7 cell-derived xenograft tumor	[59]
Stomach cancer	Apigenin treatment (30–60 mg/kg body weight/day) significantly anti-gastic cancer and anti-atrophic progression in *Helicobacter pylori*-infected Mongolian gerbils	[60]
Brain cancer	PC12 cells were pretreated with apigenin for 6 h, and then apigenin could decreased oxygen and glucose deprivation/reperfusion (OGD/R)-induced neuronal injury through apigenin-triggered antioxidative and antiapoptotic activity	[61]
Cervical cancer	Apigenin reduced the HeLa cells viability, the IC50 value was 35.89 μM. Arrested at sub-G1, G1 phase, and the upregulated p21/WAF1, and p53 protein expressions	[62]
Colon cancer	Apigenin suppresses colorectal cancer migration and metastasis through inhibition of NEDD9/Src/AKT and Wnt/β-catin signaling pathway	[63,64]
Breast cancer	Apigenin combined with chrysin synergistically decreased MDA-AM-231 cell viability, increased apoptosis, and inhibited migration at 72–96 h	[65]
Prostate cancer	Apigenin (15 μM) potentiates the anticancer effect of cisplatin to inhibit CD44^+^PCa cell growth and to significantly rescue suppressed phosphorylation of AKT and PI3K, and increased the cisplatin on the cell migration inhibitory effect	[66]
Oral cancer	Apigenin (40 mM) significantly reduced HN-30 cell viability, and apigenin (2.5 mg/kg body weight) deregulated cell proliferation, apoptosis expression, and inflammatory markers in DMBA-induced hamster pouch carcinogenesis	[67,68]

## Data Availability

The datasets generated during and/or analysed during the current study are available from the corresponding author on reasonable request.

## Supplementary Materials

The following supporting information can be downloaded at: https://www.mdpi.com/article/10.3390/cancers14071824/s1, Figure S1: Apigenin structure and LCMS analysis. Figure S2: Apoptosis and cell cycle arrest induced by apigenin in cervical cancer cells; Figure S3: Apigenin regulated PI3K/AKT/mTOR signaling pathway; Figure S4: Apigenin inactivates the integrin β1-FAK signaling pathway; Figure S5: Apigenin inhibits epithelial-to-mesenchymal transitional protein expressions.
