# Supplementary material for: Anticancer Effects and Molecular Mechanisms of Apigenin in Cervical Cancer Cells
Source: Cancers (Basel). 2022 Apr 4;14(7):1824. doi: 10.3390/cancers14071824 (PMC8998024; doi:10.3390/cancers14071824)
Supplement: Supplementary file 1 [file cancers-14-01824-s001.zip › cancers-1620705-supplementary.pptx]

## Slide 1
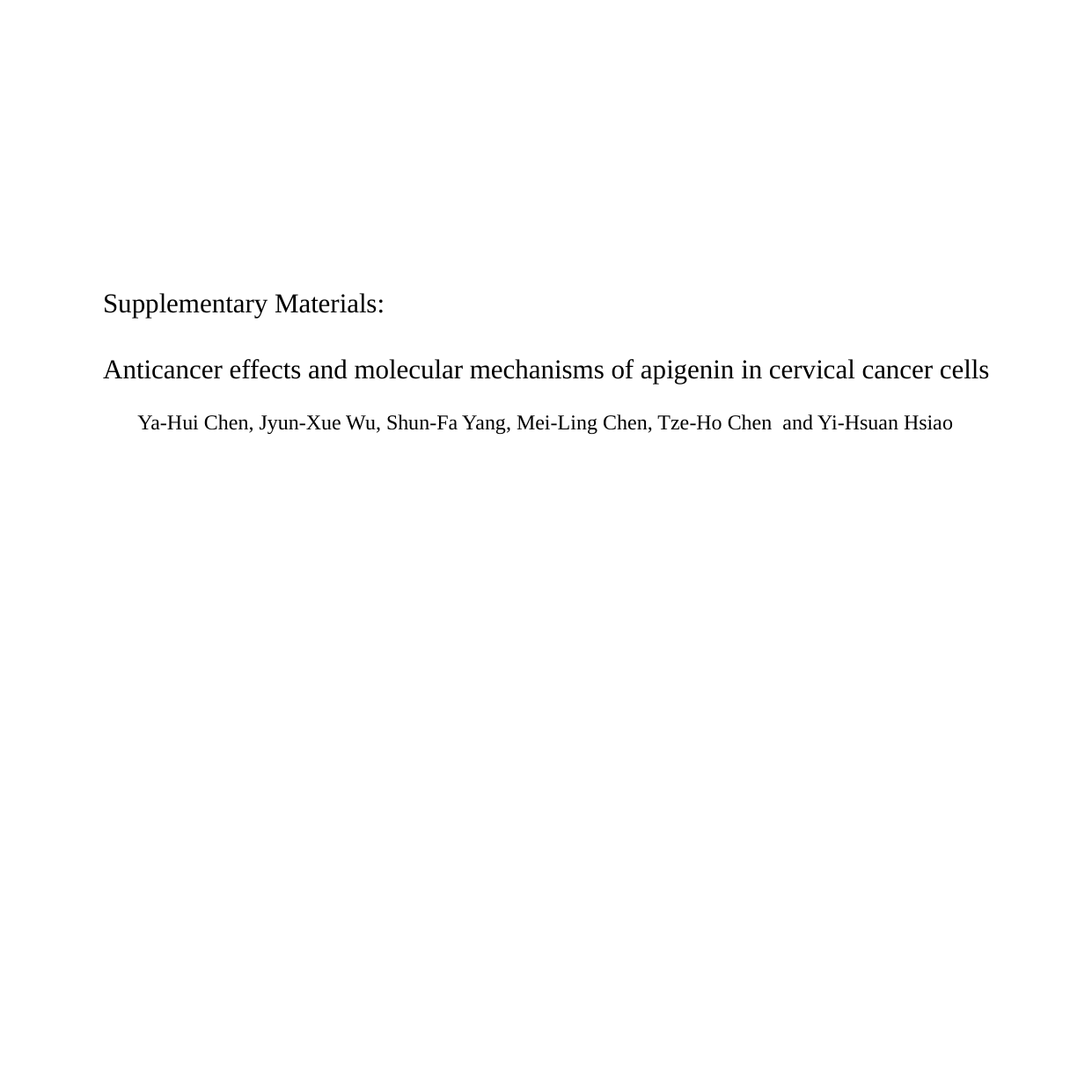

Supplementary Materials:
Anticancer effects and molecular mechanisms of apigenin in cervical cancer cells
Ya-Hui Chen, Jyun-Xue Wu, Shun-Fa Yang, Mei-Ling Chen, Tze-Ho Chen and Yi-Hsuan Hsiao

## Slide 2
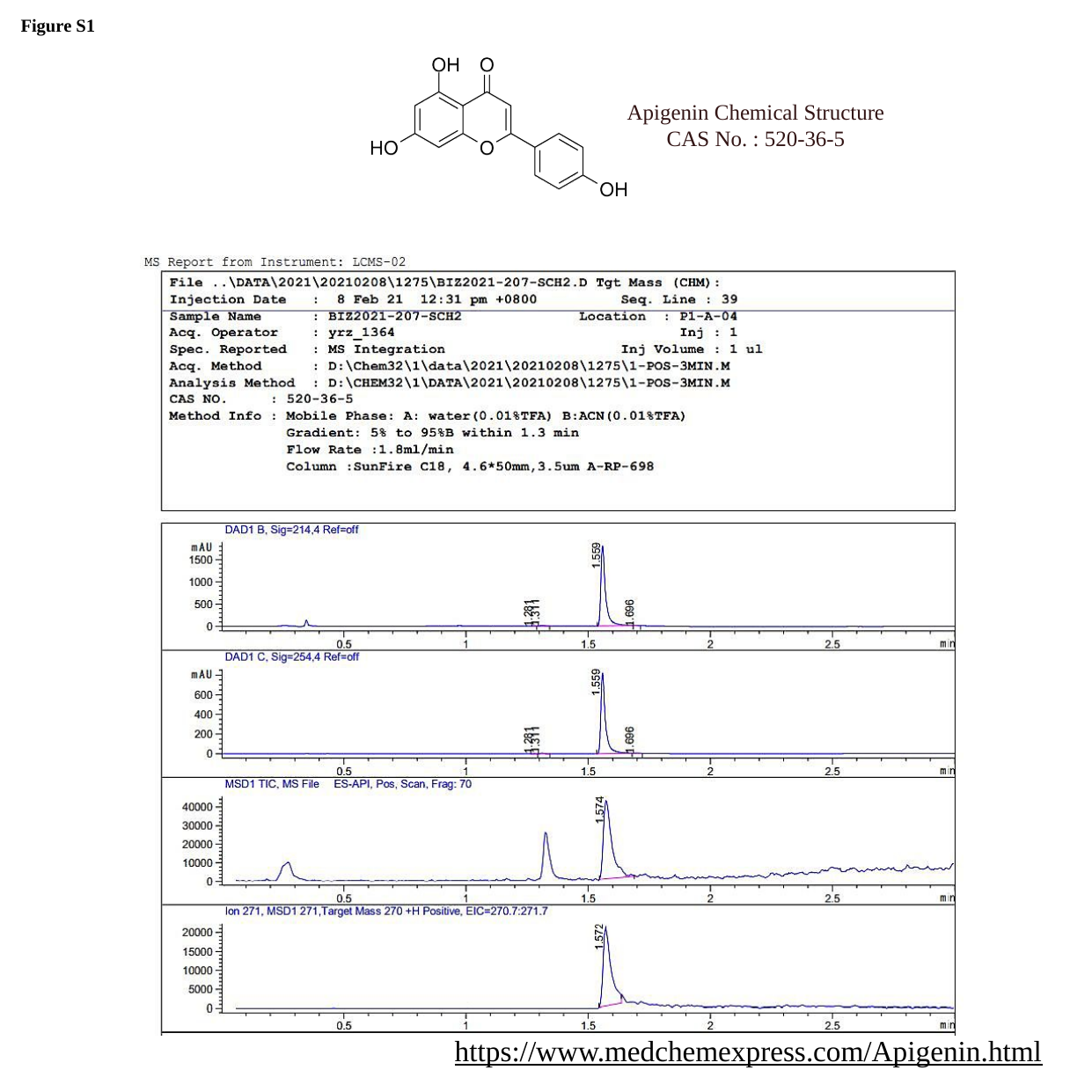

Figure S1
Apigenin Chemical Structure
CAS No. : 520-36-5
https://www.medchemexpress.com/Apigenin.html

## Slide 3
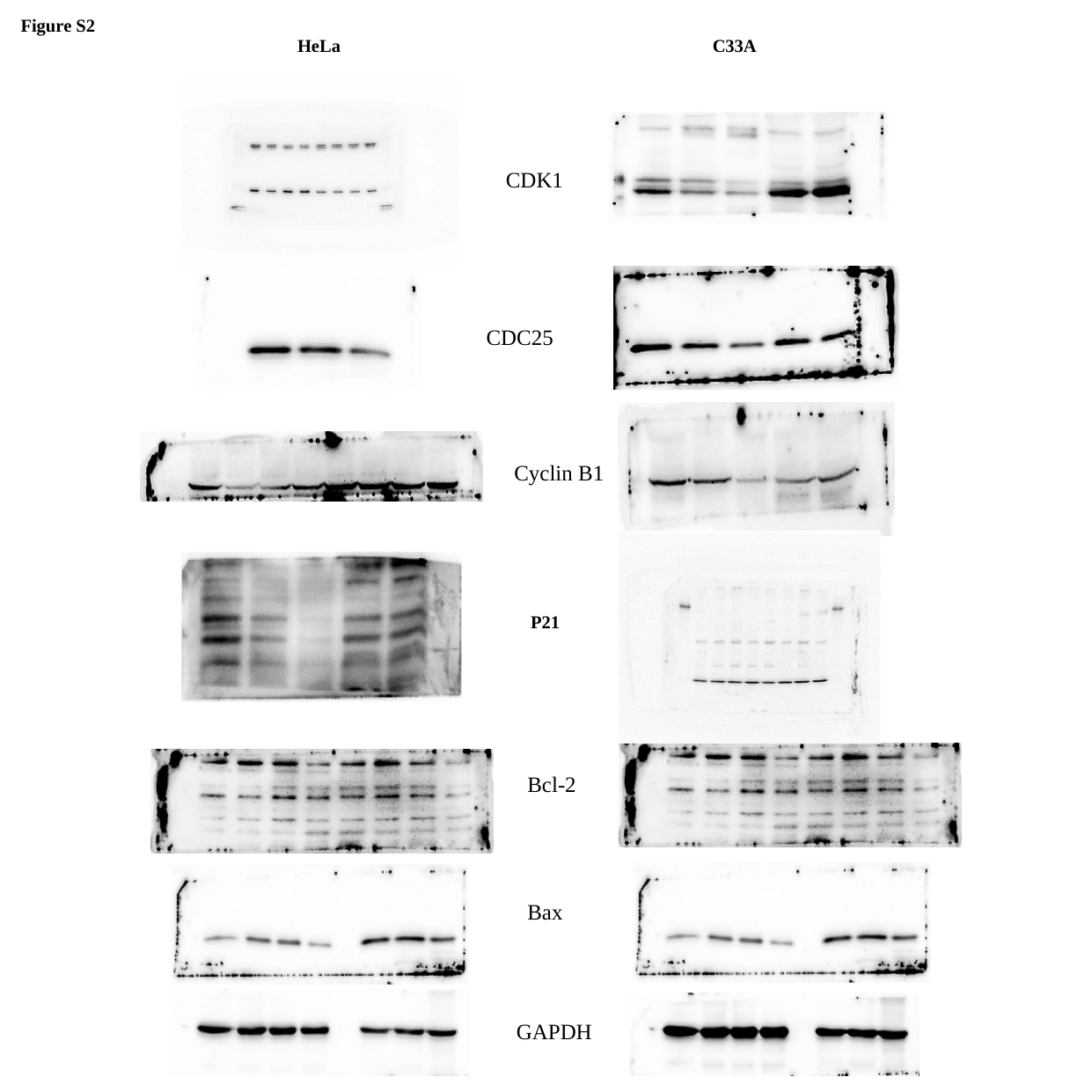

Figure S2
HeLa
C33A
CDK1
CDC25
Cyclin B1
P21
Bcl-2
Bax
GAPDH

## Slide 4
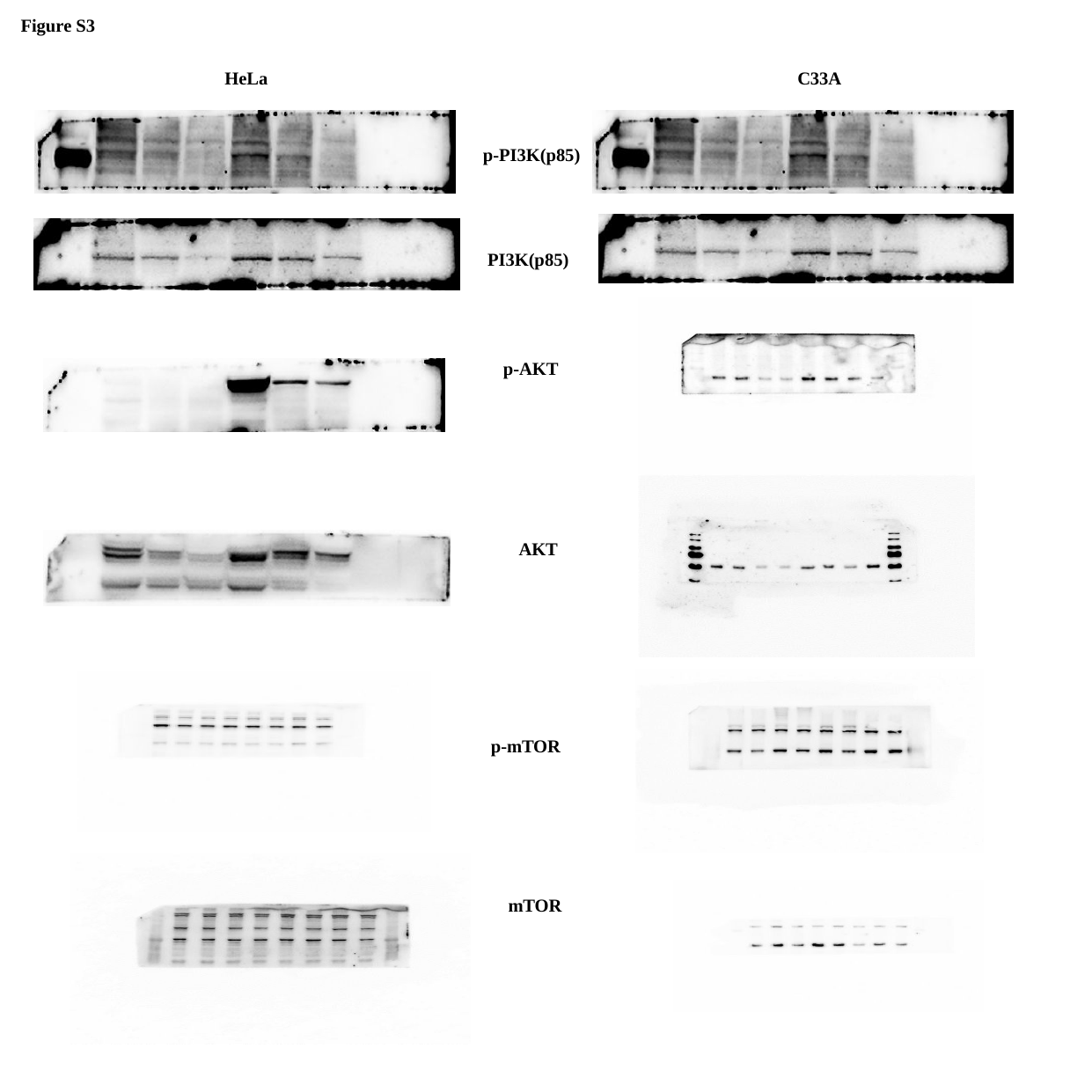

Figure S3
C33A
HeLa
p-PI3K(p85)
PI3K(p85)
p-AKT
AKT
p-mTOR
mTOR

## Slide 5
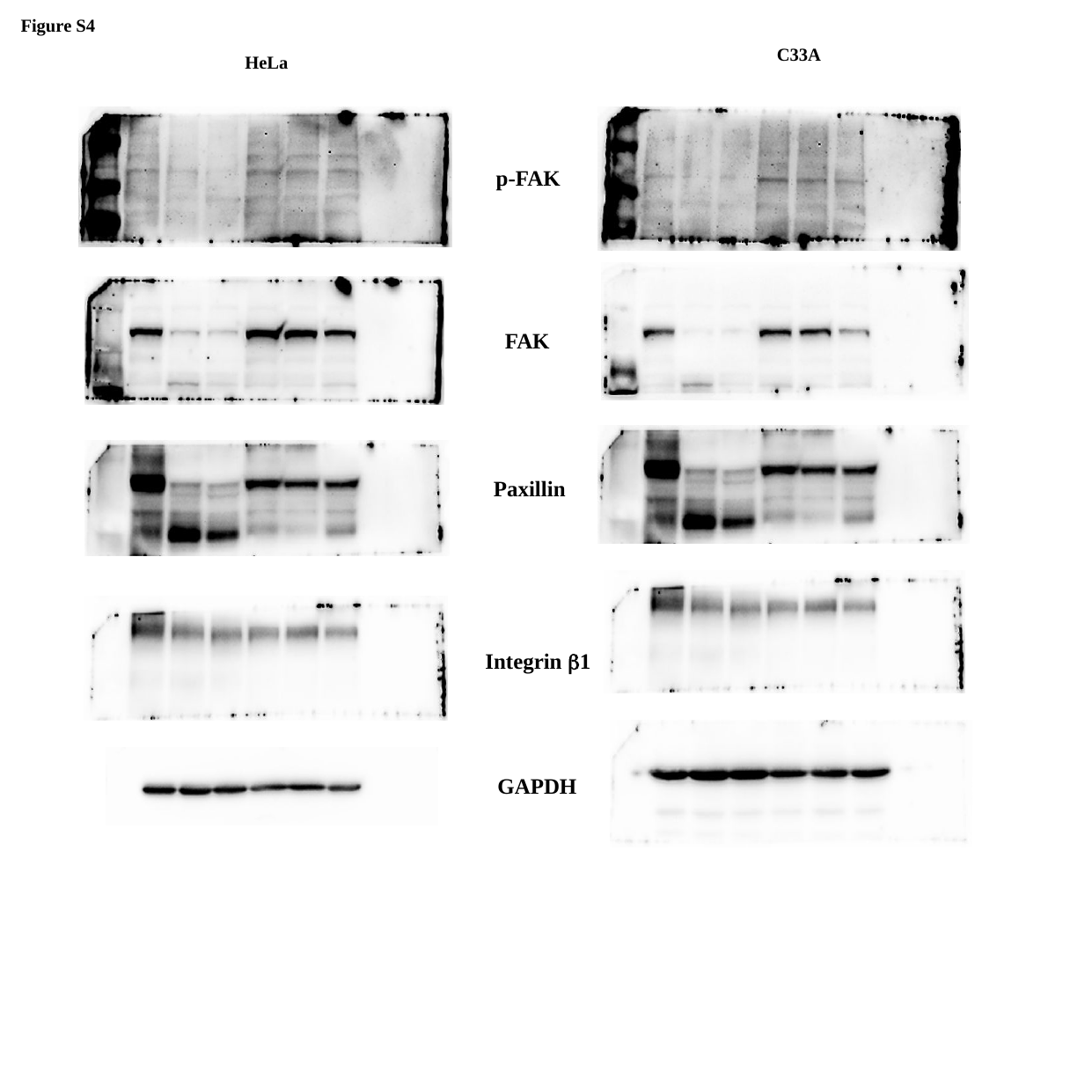

Figure S4
C33A
HeLa
p-FAK
FAK
Paxillin
Integrin β1
GAPDH

## Slide 6
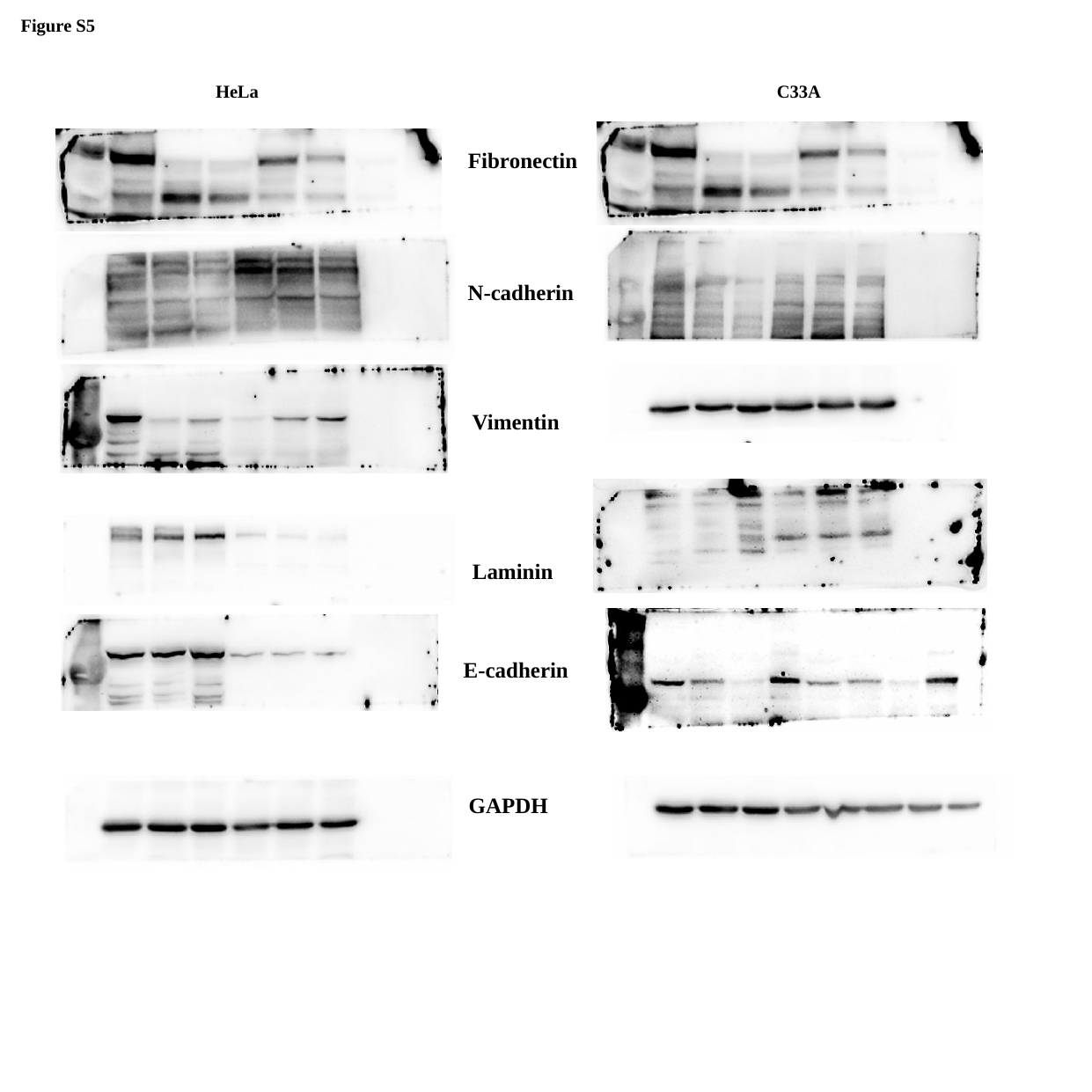

Figure S5
HeLa
C33A
Fibronectin
N-cadherin
Vimentin
Laminin
E-cadherin
GAPDH
